# 2-(4-Methyl­sulfanylphen­yl)-1*H*-benzimidazol-3-ium bromide

**DOI:** 10.1107/S1600536811000146

**Published:** 2011-01-12

**Authors:** Mohamed Ziaulla, M. N. Manjunatha, Ravish Sankolli, K. R. Nagasundara, Noor Shahina Begum

**Affiliations:** a Department of Chemistry, Bangalore University, Bangalore 560 001, India; b Solid State and Structural Chemistry Unit, Indian Institute of Science, Bangalore 560 012, India

## Abstract

In the cation of the title compound, C_14_H_13_N_2_S^+^·Br^−^, the essentially planar benzimidazole system (r.m.s. deviation = 0.0082 Å) is substituted with a 4-methyl­sulfanylphenyl ring. The dihedral angle between the benzimidazole system and the 4-methyl­sulfanylphenyl ring is 2.133 (2)°. The crystal structure is characterized by strong and highly directional inter­molecular N—H⋯Br hydrogen bonds involving the bromide ion. Moreover, C—H⋯S inter­actions result in chains of mol­ecules along the *c* axis. The supra­molecular assembly is further stabilized by π–π stacking inter­actions between the benzimidazole system and 4-methyl­sulfanylphenyl rings [centroid–centroid distance = 3.477 (4) Å].

## Related literature

For general background to benzimidazoles and their derivatives, see: Huang & Scarborough (1999 ▶); Preston (1974 ▶); Zarrinmayeh *et al.* (1998 ▶); Zhu *et al.* (2000 ▶). For related structures, see: Goker *et al.* (1995 ▶); Ozbey *et al.* (1998 ▶); Vasudevan *et al.* (1994 ▶). For hydrogen bonding, see: Bernstein *et al.* (1995 ▶); Nardelli (1983 ▶).
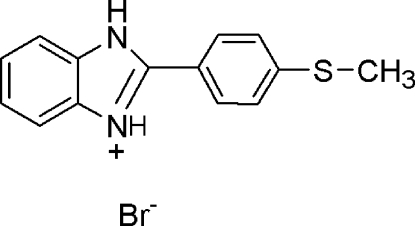


## Experimental

### 

#### Crystal data

C_14_H_13_N_2_S^+^·Br^−^
*M* *_r_* = 321.23
Monoclinic, 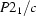

*a* = 5.3289 (2) Å
*b* = 24.0195 (12) Å
*c* = 10.9544 (5) Å
β = 100.113 (2)°
*V* = 1380.35 (11) Å^3^
*Z* = 4
Mo *K*α radiation
μ = 3.11 mm^−1^
*T* = 296 K
0.20 × 0.18 × 0.16 mm

#### Data collection

Bruker SMART APEX CCD detector diffractometer
Absorption correction: multi-scan (*SADABS*; Bruker, 1998 ▶) *T*_min_ = 0.575, *T*_max_ = 0.636
23823 measured reflections
3009 independent reflections
2273 reflections with *I* > 2σ(*I*)
*R*_int_ = 0.039

#### Refinement

*R*[*F* ^2^ > 2σ(*F* ^2^)] = 0.029
*wR*(*F* ^2^) = 0.069
*S* = 1.03
3009 reflections
215 parameters
H atoms treated by a mixture of independent and constrained refinement
Δρ_max_ = 0.35 e Å^−3^
Δρ_min_ = −0.29 e Å^−3^

### 

Data collection: *SMART* (Bruker, 1998 ▶); cell refinement: *SAINT-Plus* (Bruker, 1998 ▶); data reduction: *SAINT-Plus*; program(s) used to solve structure: *SHELXS97* (Sheldrick, 2008 ▶); program(s) used to refine structure: *SHELXL97* (Sheldrick, 2008 ▶); molecular graphics: *ORTEP-3* (Farrugia, 1997 ▶) and *CAMERON* (Watkin *et al.*, 1996) ▶; software used to prepare material for publication: *WinGX* (Farrugia, 1999 ▶).

## Supplementary Material

Crystal structure: contains datablocks global, I. DOI: 10.1107/S1600536811000146/pb2053sup1.cif

Structure factors: contains datablocks I. DOI: 10.1107/S1600536811000146/pb2053Isup2.hkl

Additional supplementary materials:  crystallographic information; 3D view; checkCIF report

## Figures and Tables

**Table 1:** Hydrogen-bond geometry (Å, °)

*D*—H⋯*A*	*D*—H	H⋯*A*	*D*⋯*A*	*D*—H⋯*A*
N1—H1*N*⋯Br1	0.74 (2)	2.51 (2)	3.247 (2)	171 (2)
N2—H2*N*⋯Br1^i^	0.77 (3)	2.50 (2)	3.231 (2)	159
C5—H5⋯S1^ii^	0.97 (3)	2.98 (3)	3.736 (3)	135

Symmetry codes: (i) 
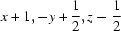
; (ii) 
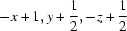
.

**Fig. 1.**
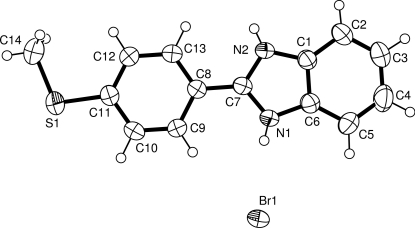
ORTEP (Farrugia, 1997) view of the title compound, showing 50% probability ellipsoids and the atom numbering scheme.

**Fig. 2.**
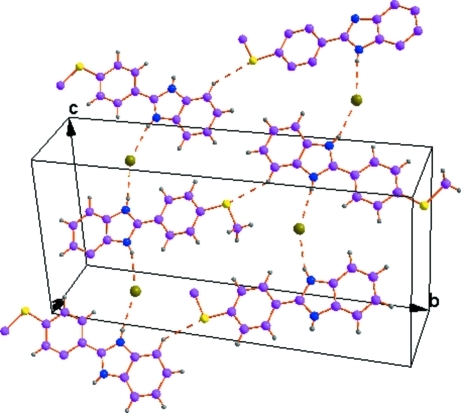
A unit cell packing of the title compound showing intermolecular interactions with dotted lines. H-atoms not involved in hydrogen bonding have been excluded.

## supplementary crystallographic information

### Comment

Benzimidazoles and their derivatives exhibit a number of important pharmacological properties, such as antihistaminic, anti-ulcerative, antiallergic, and antipyretic. In addition, benzimidazole derivatives are effective against the human cytomegalo virus (HCMV) (Zhu *et al.*, 2000) and are also efficient selective neuropeptide Y Y1 receptor antagonists (Zarrinmayeh *et al.*, 1998). Most of the described methods for the synthesisof benzimidazoles make use of volatile organic solvents and involve solid-phase synthesis *via o*-nitroanilines (Preston *et al.*, 1974; Huang *et al.*, 1999) or the condensation of *o*-phenylenediamines with carboxylic acid derivatives, aldehydes and aryl halides. In the title compound, there is one benzimidazole thiomethyl phenyl cation and one Br^-^ anion in the asymmetric unit. The expected proton transfer from HBr to benzimidazole thiomethyl phenyl occurs at atom N1 of the benzimidazole ring. Consequently,atom N1 shows quaternary character and bears a positive charge. In the molecule, the benzimidazole and thiomethyl phenyl rings are planar inclined at an dihedral angle 2.133 (2)° between them. The molecular structure is primarily stabilized by strong intramolecular N—H···Br hydrogen bond. The bond lengths and angles for the benzimidazole moiety of the molecule are in good agreement, within experimental errors, with those observed in other benzimidazole derivatives (Goker *et al.*, 1995; Ozbey *et al.*, 1998; Vasudevan *et al.*,1994).Further, the crystal structure is stabilized by intermolecular interactions into three dimensional framework structure by the combination of C—H···S and N—H···Br. The C—H···S and N—H···Br interactions together generates tetramers linking the molecules into chain like pattern along crystallographic *c*-axis. Additionally,the supramolecular assembly is further stabilized by π-π-stacking interactions between the benzimidazole and thiomethyl phenyl rings. The C3—C10 (*x*, 0.5 - *y*, 1/2 + *z*) disposed at a distance of 3.477 (4) Å.

### Experimental

A ethanol solution (20 ml) of zinc bromide (2.25 mg, 1.0 mmol) was treated with 2-(*p*-thiomethylphenyl)benzimidazole (4.80 mg, 2.0 mmol) in ethanol (20 ml). The mixture was then treated with 48% HBr (2–3 ml) followed by liquid Br2 (2–3 ml). The mixture was refluxed for 6 hrs on a steam bath filtered and allowed to stand at room temperature for two days. Coloured crystals separated and these were washed with ethanol and dried. (yield 4.00 mg; 83%).

### Crystal data

**Table:**

C_14_H_13_N_2_S^+^·Br^−^	*F*(000) = 648
*M**_r_* = 321.23	*D*_x_ = 1.546 Mg m^−^^3^
Monoclinic, *P*2_1_/*c*	Mo *K*α radiation, λ = 0.71073 Å
Hall symbol: -P 2ybc	Cell parameters from 3009 reflections
*a* = 5.3289 (2) Å	θ = 1.7–27.0°
*b* = 24.0195 (12) Å	µ = 3.11 mm^−^^1^
*c* = 10.9544 (5) Å	*T* = 296 K
β = 100.113 (2)°	Block, yellow
*V* = 1380.35 (11) Å^3^	0.20 × 0.18 × 0.16 mm
*Z* = 4	

### Data collection

**Table:**

Bruker SMART APEX CCD detector diffractometer	3009 independent reflections
Radiation source: Enhance (Mo) X-ray Source	2273 reflections with *I* > 2σ(*I*)
graphite	*R*_int_ = 0.039
ω scans	θ_max_ = 27.0°, θ_min_ = 1.7°
Absorption correction: multi-scan Bruker Kappa *APEX*	*h* = −6→6
*T*_min_ = 0.575, *T*_max_ = 0.636	*k* = −30→30
23823 measured reflections	*l* = −13→13

### Refinement

**Table:**

Refinement on *F*^2^	Primary atom site location: structure-invariant direct methods
Least-squares matrix: full	Secondary atom site location: difference Fourier map
*R*[*F*^2^ > 2σ(*F*^2^)] = 0.029	Hydrogen site location: inferred from neighbouring sites
*wR*(*F*^2^) = 0.069	H atoms treated by a mixture of independent and constrained refinement
*S* = 1.03	*w* = 1/[σ^2^(*F*_o_^2^) + (0.0338*P*)^2^ + 0.3109*P*] where *P* = (*F*_o_^2^ + 2*F*_c_^2^)/3
3009 reflections	(Δ/σ)_max_ = 0.001
215 parameters	Δρ_max_ = 0.35 e Å^−^^3^
0 restraints	Δρ_min_ = −0.29 e Å^−^^3^

### Special details

**Table:**

Geometry. All e.s.d.'s (except the e.s.d. in the dihedral angle between two l.s. planes) are estimated using the full covariance matrix. The cell e.s.d.'s are taken into account individually in the estimation of e.s.d.'s in distances, angles and torsion angles; correlations between e.s.d.'s in cell parameters are only used when they are defined by crystal symmetry. An approximate (isotropic) treatment of cell e.s.d.'s is used for estimating e.s.d.'s involving l.s. planes.
Refinement. Refinement of *F*^2^ against ALL reflections. The weighted *R*-factor *wR* and goodness of fit *S* are based on *F*^2^, conventional *R*-factors *R* are based on *F*, with *F* set to zero for negative *F*^2^. The threshold expression of *F*^2^ > σ(*F*^2^) is used only for calculating *R*-factors(gt) *etc*. and is not relevant to the choice of reflections for refinement. *R*-factors based on *F*^2^ are statistically about twice as large as those based on *F*, and *R*- factors based on ALL data will be even larger.

### Fractional atomic coordinates and isotropic or equivalent isotropic displacement parameters (Å^2^)

**Table:**

	*x*	*y*	*z*	*U*_iso_*/*U*_eq_	
Br1	0.27225 (4)	0.295277 (10)	0.33608 (2)	0.04982 (10)	
S1	0.57379 (16)	−0.00128 (3)	0.21720 (8)	0.0710 (2)	
N1	0.6485 (4)	0.28057 (8)	0.13508 (19)	0.0421 (5)	
N2	0.9186 (4)	0.25577 (8)	0.01982 (17)	0.0408 (4)	
C1	0.9109 (4)	0.31328 (10)	0.0161 (2)	0.0415 (5)	
C2	1.0437 (5)	0.35189 (11)	−0.0424 (2)	0.0555 (6)	
C3	0.9910 (6)	0.40696 (12)	−0.0233 (3)	0.0644 (7)	
C4	0.8149 (6)	0.42307 (12)	0.0504 (3)	0.0659 (8)	
C5	0.6847 (5)	0.38481 (11)	0.1084 (3)	0.0555 (6)	
C6	0.7381 (4)	0.32913 (10)	0.0906 (2)	0.0430 (5)	
C7	0.7610 (4)	0.23676 (9)	0.09243 (19)	0.0387 (5)	
C8	0.7198 (4)	0.17861 (9)	0.1213 (2)	0.0391 (5)	
C9	0.5449 (5)	0.16389 (11)	0.1960 (2)	0.0516 (6)	
C10	0.5058 (5)	0.10930 (11)	0.2220 (2)	0.0558 (6)	
C11	0.6392 (5)	0.06710 (10)	0.1762 (2)	0.0453 (5)	
C12	0.8139 (6)	0.08164 (11)	0.1013 (3)	0.0586 (7)	
C13	0.8530 (5)	0.13648 (11)	0.0746 (3)	0.0548 (7)	
C14	0.7734 (8)	−0.04379 (14)	0.1398 (4)	0.0721 (9)	
H1N	0.562 (5)	0.2802 (10)	0.181 (2)	0.042 (7)*	
H9	0.464 (5)	0.1907 (12)	0.233 (2)	0.062 (8)*	
H2N	0.991 (5)	0.2357 (11)	−0.017 (2)	0.049 (8)*	
H12	0.909 (5)	0.0539 (11)	0.070 (2)	0.065 (8)*	
H14C	0.736 (6)	−0.0776 (16)	0.159 (3)	0.086 (11)*	
H14B	0.947 (7)	−0.0359 (13)	0.169 (3)	0.090 (11)*	
H5	0.567 (5)	0.3946 (12)	0.163 (2)	0.075 (9)*	
H4	0.781 (6)	0.4619 (13)	0.063 (3)	0.085 (10)*	
H2	1.159 (5)	0.3398 (11)	−0.092 (2)	0.058 (7)*	
H13	0.975 (5)	0.1462 (12)	0.022 (3)	0.079 (9)*	
H10	0.389 (5)	0.0996 (11)	0.276 (2)	0.064 (7)*	
H3	1.080 (5)	0.4350 (12)	−0.060 (2)	0.070 (8)*	
H14A	0.736 (6)	−0.0374 (14)	0.054 (3)	0.097 (12)*	

### Atomic displacement parameters (Å^2^)

**Table:**

	*U*^11^	*U*^22^	*U*^33^	*U*^12^	*U*^13^	*U*^23^
Br1	0.05371 (15)	0.05390 (17)	0.04775 (15)	0.00057 (11)	0.02515 (10)	0.00017 (11)
S1	0.0965 (6)	0.0390 (4)	0.0891 (5)	−0.0055 (3)	0.0480 (4)	0.0060 (3)
N1	0.0446 (11)	0.0397 (11)	0.0476 (11)	−0.0021 (8)	0.0233 (9)	−0.0012 (9)
N2	0.0441 (10)	0.0396 (11)	0.0436 (11)	−0.0006 (9)	0.0212 (9)	−0.0029 (9)
C1	0.0442 (12)	0.0399 (12)	0.0414 (12)	−0.0043 (10)	0.0105 (10)	−0.0003 (10)
C2	0.0593 (16)	0.0521 (16)	0.0598 (15)	−0.0093 (12)	0.0233 (13)	0.0045 (13)
C3	0.0741 (19)	0.0474 (16)	0.0755 (18)	−0.0129 (14)	0.0231 (15)	0.0080 (14)
C4	0.077 (2)	0.0386 (16)	0.082 (2)	−0.0056 (13)	0.0140 (16)	−0.0001 (14)
C5	0.0626 (16)	0.0411 (15)	0.0655 (16)	0.0024 (12)	0.0185 (13)	−0.0077 (13)
C6	0.0433 (12)	0.0414 (13)	0.0450 (12)	−0.0031 (10)	0.0099 (9)	−0.0011 (10)
C7	0.0380 (11)	0.0407 (13)	0.0392 (11)	−0.0013 (10)	0.0121 (9)	0.0001 (10)
C8	0.0400 (12)	0.0394 (13)	0.0392 (11)	−0.0015 (10)	0.0109 (9)	0.0009 (10)
C9	0.0621 (16)	0.0380 (13)	0.0626 (15)	0.0046 (11)	0.0331 (13)	0.0005 (12)
C10	0.0620 (16)	0.0484 (15)	0.0665 (16)	−0.0018 (12)	0.0377 (13)	0.0054 (13)
C11	0.0512 (13)	0.0386 (13)	0.0483 (13)	−0.0036 (10)	0.0147 (10)	0.0016 (10)
C12	0.0705 (18)	0.0389 (15)	0.0762 (18)	0.0019 (12)	0.0400 (15)	−0.0019 (13)
C13	0.0615 (16)	0.0436 (15)	0.0689 (16)	0.0011 (12)	0.0377 (14)	−0.0004 (12)
C14	0.091 (3)	0.0407 (18)	0.089 (3)	0.0024 (16)	0.028 (2)	−0.0003 (16)

### Geometric parameters (Å, °)

**Table:**

S1—C11	1.754 (2)	C5—C6	1.388 (3)
S1—C14	1.791 (4)	C5—H5	0.97 (3)
N1—C7	1.335 (3)	C7—C8	1.457 (3)
N1—C6	1.381 (3)	C8—C13	1.384 (3)
N1—H1N	0.74 (3)	C8—C9	1.390 (3)
N2—C7	1.334 (3)	C9—C10	1.365 (4)
N2—C1	1.382 (3)	C9—H9	0.91 (3)
N2—H2N	0.77 (3)	C10—C11	1.382 (3)
C1—C6	1.386 (3)	C10—H10	0.96 (3)
C1—C2	1.390 (3)	C11—C12	1.389 (3)
C2—C3	1.376 (4)	C12—C13	1.373 (4)
C2—H2	0.93 (3)	C12—H12	0.94 (3)
C3—C4	1.395 (4)	C13—H13	0.97 (3)
C3—H3	0.95 (3)	C14—H14C	0.87 (4)
C4—C5	1.373 (4)	C14—H14B	0.94 (3)
C4—H4	0.96 (3)	C14—H14A	0.94 (3)
			
C11—S1—C14	104.57 (15)	N2—C7—C8	126.29 (19)
C7—N1—C6	109.73 (19)	N1—C7—C8	125.82 (18)
C7—N1—H1N	127.0 (19)	C13—C8—C9	118.2 (2)
C6—N1—H1N	123.0 (19)	C13—C8—C7	120.93 (19)
C7—N2—C1	109.94 (18)	C9—C8—C7	120.9 (2)
C7—N2—H2N	121.6 (19)	C10—C9—C8	120.6 (2)
C1—N2—H2N	128.3 (19)	C10—C9—H9	118.9 (17)
N2—C1—C6	106.04 (19)	C8—C9—H9	120.3 (17)
N2—C1—C2	131.7 (2)	C9—C10—C11	121.5 (2)
C6—C1—C2	122.2 (2)	C9—C10—H10	120.0 (16)
C3—C2—C1	115.9 (3)	C11—C10—H10	118.4 (16)
C3—C2—H2	124.1 (16)	C10—C11—C12	118.1 (2)
C1—C2—H2	120.0 (16)	C10—C11—S1	117.15 (18)
C2—C3—C4	122.1 (3)	C12—C11—S1	124.79 (19)
C2—C3—H3	119.2 (17)	C13—C12—C11	120.6 (2)
C4—C3—H3	118.7 (17)	C13—C12—H12	119.2 (17)
C5—C4—C3	121.9 (3)	C11—C12—H12	120.2 (17)
C5—C4—H4	117.2 (19)	C12—C13—C8	121.1 (2)
C3—C4—H4	121.0 (19)	C12—C13—H13	120.1 (17)
C4—C5—C6	116.5 (3)	C8—C13—H13	118.9 (18)
C4—C5—H5	123.9 (18)	S1—C14—H14C	104 (2)
C6—C5—H5	119.5 (18)	S1—C14—H14B	111 (2)
N1—C6—C1	106.4 (2)	H14C—C14—H14B	111 (3)
N1—C6—C5	132.2 (2)	S1—C14—H14A	110 (2)
C1—C6—C5	121.4 (2)	H14C—C14—H14A	112 (3)
N2—C7—N1	107.9 (2)	H14B—C14—H14A	109 (3)
			
C7—N2—C1—C6	−0.2 (3)	C6—N1—C7—C8	178.60 (19)
C7—N2—C1—C2	177.8 (3)	N2—C7—C8—C13	1.4 (3)
N2—C1—C2—C3	−178.5 (2)	N1—C7—C8—C13	−178.0 (2)
C6—C1—C2—C3	−0.8 (4)	N2—C7—C8—C9	−178.3 (2)
C1—C2—C3—C4	0.0 (4)	N1—C7—C8—C9	2.2 (3)
C2—C3—C4—C5	0.3 (5)	C13—C8—C9—C10	−0.2 (4)
C3—C4—C5—C6	0.3 (4)	C7—C8—C9—C10	179.5 (2)
C7—N1—C6—C1	0.8 (3)	C8—C9—C10—C11	0.6 (4)
C7—N1—C6—C5	−179.2 (3)	C9—C10—C11—C12	−0.7 (4)
N2—C1—C6—N1	−0.4 (2)	C9—C10—C11—S1	179.5 (2)
C2—C1—C6—N1	−178.6 (2)	C14—S1—C11—C10	178.8 (2)
N2—C1—C6—C5	179.6 (2)	C14—S1—C11—C12	−1.0 (3)
C2—C1—C6—C5	1.4 (4)	C10—C11—C12—C13	0.4 (4)
C4—C5—C6—N1	178.9 (3)	S1—C11—C12—C13	−179.8 (2)
C4—C5—C6—C1	−1.1 (4)	C11—C12—C13—C8	−0.1 (5)
C1—N2—C7—N1	0.7 (3)	C9—C8—C13—C12	−0.1 (4)
C1—N2—C7—C8	−178.83 (19)	C7—C8—C13—C12	−179.8 (2)
C6—N1—C7—N2	−0.9 (3)		

### Hydrogen-bond geometry (Å, °)

**Table:**

*D*—H···*A*	*D*—H	H···*A*	*D*···*A*	*D*—H···*A*
N1—H1N···Br1	0.74 (2)	2.51 (2)	3.247 (2)	171 (2)
N2—H2N···Br1^i^	0.77 (3)	2.50 (2)	3.231 (2)	159
C5—H5···S1^ii^	0.97 (3)	2.98 (3)	3.736 (3)	135

Symmetry codes:  (i) *x*+1, −*y*+1/2, *z*−1/2; (ii) −*x*+1, *y*+1/2, −*z*+1/2.
